# Patterns of Leaf and Fruit Morphological Variation in Marginal Populations of *Acer tataricum* L. subsp. *tataricum*

**DOI:** 10.3390/plants13020320

**Published:** 2024-01-21

**Authors:** Igor Poljak, Antonio Vidaković, Luka Benić, Katarina Tumpa, Marilena Idžojtić, Zlatko Šatović

**Affiliations:** 1 Institute of Forest Genetics, Dendrology and Botany, Faculty of Forestry and Wood Technology, University of Zagreb, Svetošimunska cesta 23, HR-10000 Zagreb, Croatia; ipoljak@sumfak.unizg.hr (I.P.); avidakovi@sumfak.unizg.hr (A.V.); lbenic@sumfak.unizg.hr (L.B.); ktumpa@sumfak.unizg.hr (K.T.); midzojtic@sumfak.unizg.hr (M.I.); 2 Department for Seed Science and Technology, Faculty of Agriculture, University of Zagreb, Svetošimunska cesta 25, HR-10000 Zagreb, Croatia; 3 Centre of Excellence for Biodiversity and Molecular Plant Breeding, Svetošimunska cesta 25, HR-10000 Zagreb, Croatia

**Keywords:** leading-edge populations, plant variation, plant morphology, morphometric analysis, geographical differentiation, environmental differentiation, population variability

## Abstract

Marginal populations are usually smaller and more isolated and grow in less favourable conditions than those at the distribution centre. The variability of these populations is of high importance, as it can support the adaptations needed for the conditions that they grow in. In this research, the morphological variability of eight Tatar maple (*Acer tataricum* L. subsp. *tataricum*) populations was analysed. Tatar maple is an insect-pollinated and wind-dispersed shrub/tree, whose northwestern distribution edge is in southeastern Europe. Morphometric methods were used to analyse the variability of the populations using leaf and fruit morphology. The research revealed significant differences between and within populations. Furthermore, differences in the distribution of the total variability were noted, which suggest that different evolutionarily factors affect different plant traits. Correlation analysis confirmed a weak dependency between the vegetative and generative traits. In addition, no evidence was found for the presence of isolation by environment (IBE). However, the Mantel test for isolation by distance (IBD) was significant for the leaf morphometric traits and non-significant for the fruit morphometric traits. Being the marginal leading-edge populations, they are younger and were less likely to have had time for adaptation to local environments, which would have resulted in the development of IBE. Overall, edge populations of Tatar maple were characterised by great morphological variability, which helps these populations in their response to the intensive selective pressures they face in their environment.

## 1. Introduction

A certain degree of morphological variability of vegetative and generative organs is characteristic of plant species [1]. This variability is, to some extent, conditioned by genetic variability [2] but also by environmental heterogeneity [1,3]. Many authors have determined the influence of various environmental conditions on leaf morphology, including temperature [4,5], precipitation [6,7] and insolation gradients [8,9]. Other studies confirm that not only are the leaves prone to environmentally induced morphological changes, but the fruits are as well [10,11]. However, most of the research on morphological variability to date has been carried out on either leaves or fruits, rarely both [12,13].

In wind-pollinated species, unhindered gene flow usually occurs, which has a homogenizing effect on a species’ genetic and morphological variability [14]. Therefore, differentiation between populations is expected to be low, as confirmed for *Acer campestre* L. [15], *A. monspessulanum* L. [16] and *Pinus sylvestris* L. [14]. This, however, does not imply a low diversity within populations; on the contrary, it is usually significantly higher than between populations [17]. Variability, both genetic and morphological, is crucial for adaptation, biodiversity and speciation [18] and is under the influence of several evolutionary processes, including gene flow, genetic drift and natural selection. There are two well-known patterns that explain divergence among populations: isolation by distance (IBD) and isolation by environment (IBE). The isolation-by-distance model indicates that differentiation between populations increases with geographical distance [19], arising from limited gene flow and the presence of genetic drift. The original model of isolation by distance presented by Wright [20] showed how population structure is generated by limits on parent-offspring dispersal distances and that this process is expected to be more pronounced as population size decreases. On the other hand, isolation by environment (IBE) explains genetic and morphological differentiation through environmental differences between populations, where genetic/morphological and environmental distances are positively correlated, independent of geographical distance [19,21]. However, these patterns are not exclusive, as confirmed in the study by Sexton et al. [22], indicating that differences among populations can follow both IBD and IBE patterns.

Populations that are particularly affected by limiting gene flow and other selective pressures are marginal populations [23,24]. Therefore, marginal populations are likely to have increased genetic differentiation [25,26]. As a result, they are characterised by the ability to adapt to suboptimal environments, which makes them important from the evolutionary point of view [23]. Furthermore, this adaptability marks them as highly important for the long-term conservation of genetic diversity of species [27]. With climate change in mind, marginal populations will represent potential gene pools for the expansion and shift of the species away from the drought in the south towards the newly formed, favorable habitats to the north [28]. They are usually characterised by greater demographic stochasticity and differentiation whilst simultaneously being the youngest and with the lowest regional diversity [25,27].

Although research into the plant species marginal populations has been conducted from a genetics point of view [28,29,30,31], such research from the morphological point of view is sparse [32]. In this study, therefore, we aimed at using the morphological characteristics to gain insight into the diversity of marginal populations. As a model species, the Tatar maple (*Acer tataricum* L. subsp. *tataricum*, Sapindaceae) was selected, which reaches its northern and northwestern ranges’ limits in southeastern Europe. It is a slow-growing, small tree that reaches a height of 5 to 7 m, often with multiple or forked trunks. Flowers are scented and white and pollinated by insects. The fruits are samaras, winged nutlets that ripen in late summer or early autumn. The species is predominantly found in the lowlands or the colline zone, with an overall range between 300 and 1700 m a.s.l. Although native to warm and dry climates, it is successfully cultivated across Europe and Asia, although the trees are susceptible to frost [33].

Leaf and fruit morphological traits of eight populations of Tatar maple were analysed, with the aim of investigating the following: (1) morphological intra- and interpopulation diversity and (2) the level of phenotypical variability in the context of isolation by distance (IBD) and isolation by environment (IBE).

## 2. Materials and Methods

### 2.1. Plant Material

**Figure 1:**
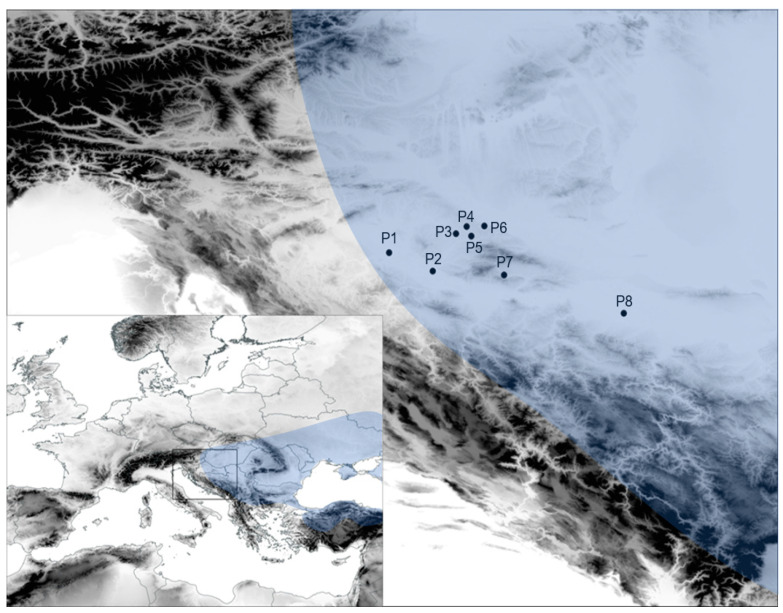
Locations of the eight sampled Tatar maple (*Acer tataricum* L. subsp. *tataricum*) populations. Populations: P1—Odransko polje; P2—Lipovljani; P3—Veliki Grđevac; P4—Mali Grđevac; P5—Grubišno Polje; P6—Virovitica; P7—Požega; P8—Županja. The blue area represents the natural habitat of Tatar maple according to Bartha [33].

In the research, eight populations of Tatar maple were sampled (Figure 1, Table S1). Samples were collected only from fully developed individuals 5 to 7 m tall and about 30 years old. All analysed populations grew in homogeneous pedological conditions. According to Bartha [34], Tatar maple fruits ripen in late August and early September, which is why we conducted sample collection in late September, to make sure that the samaras were fully ripened and that the leaf fall had not started. Leaf and fruit samples were collected simultaneously, 20 leaves and samaras per individual plant, with a total of 10 to 15 individuals per population. For sampling, well-lit branches were selected. The plant material was stored in airtight bags in the field and herbarised the same day upon arrival in the laboratory, following the protocol of plant collecting and documentation by Simpson [35]. Samples were herbarised between newspaper sheets in the herbarium of the Faculty of Forestry and Wood Technology in Zagreb (Herbarium DEND).

### 2.2. Leaf and Fruit Morphometric Analysis

**Figure 2:**
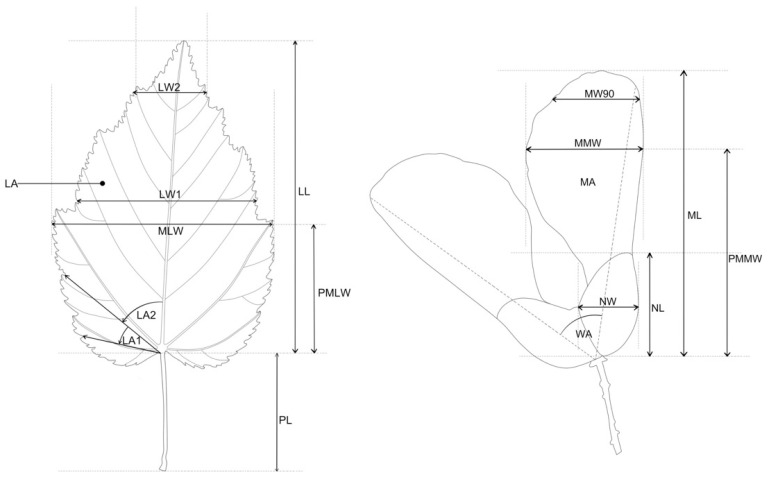
Measured leaf and fruit traits: LA—leaf area; LL—leaf length; MLW—maximum leaf width; PMLW—leaf length, measured from the leaf base to the point of maximum leaf width; LW1—leaf blade width at 50% of leaf blade length; LW2—leaf blade width at 90% of leaf blade length; PL—petiole length; LA1—angle closed by the main leaf vein (the centre of the leaf blade) and the line connecting the leaf blade base to a set point on the leaf margin at 10% of the total leaf blade length; and LA2—angle closed by the main leaf vein (the centre of the leaf blade) and the line connecting the leaf blade base to a set point on the leaf margin at 25% of the total leaf blade length; MA—mericarp area; ML—mericarp length; MMW—maximum mericarp width; PMMW—mericarp length, measured from the mericarp base to the point of maximum mericarp width; NW90—mericarp width at 90% of the fruit length; NL—nut length; NW—nut width; WA—wing angle.

After being herbarised and left to fully dry, the leaves were scanned with a MICROTEK ScanMaker 4800 (Microtek International, Inc., Hsinchu, Taiwan). The created scans were used in the WinFolia [36] morphometric analysis program by using the in-program parameters as follows: leaf area (LA); leaf length (LL); maximum leaf width (MLW); leaf length, measured from the leaf base to the point of maximum leaf width (PMLW); leaf blade width at 50% (LW1) and 90% of leaf blade length (LW2); and petiole length (PL). In addition, three leaf shape measurements were conducted, describing the leaf shape by angles enclosed by the main leaf vein (the centre of the leaf blade) and the line connecting the leaf blade base to a set point on the leaf margin, at 10% (LA1) and 25% (LA2) of the total leaf blade length, as well as the shape of the leaf blade itself (FC) (Figure 2).

Maple fruits, i.e., the mericarps, were also scanned and measured using the WinFolia program [36], with the following traits measured: mericarp area (MA); mericarp length (ML); maximum mericarp width (MMW); length of the mericarp, measured from the base to the point of maximum width (PMMW); width of the mericarp at 90% of the mericarp’s length (MW90); nut length (NL); and nut width (NW). For fruits, an interactive measurement was used for the angle enclosed by the wings (WA) (Figure 2).

### 2.3. Statistical Analysis

Descriptive statistics were calculated for each individual trait and for each population, with the goal of revealing the overall range of their variability [37]. In addition, Pearson correlation coefficients were calculated between all leaf and fruit traits. Analysis of variance was used to determine the variability between the studied populations, as well as between trees/shrubs within the populations. The “tree/shrub” factor was nested within the “population” factor. Descriptive statistics and analysis of variance were carried out using the STATISTICA software package Version 13 [38].

The Mantel test was used to evaluate the correlations between the morphometric, geographic, and environmental data [37]. In this study, four dissimilarity matrices were calculated in order to describe the differences between the analysed populations: (1) leaf morphometric differences as squared Mahalanobis distances between the pairs of populations; (2) fruit morphometric differences as squared Mahalanobis distances between the pairs of populations; (3) environmental distances as the Euclidian distances between the population means for the first three PCs of the principal component analysis; and (4) geographic distance from the latitude and longitude of the sampling site. The significance level was assessed after 10,000 permutations as implemented in NTSYS-pc Ver. 2.21L [39].

## 3. Results

### 3.1. Correlations

A total of 93 statistically significant correlations were observed between the tested pairs of leaf and fruit morphological traits, none of which were of a negative character. The strength of a relationship based on the *r* value was classified according to Sokal and Rohlf [37] as follows: none or very weak (*r* < 0.3); weak (0.3 < *r* < 0.5); moderate (0.5 < *r* < 0.7); and strong (*r* > 0.7). Out of the total 93 correlations, only 22 demonstrated *r* values larger than 0.7.

When leaf characteristics are considered (Table S2), 39 significant correlations were detected, among which 15 were strong. The traits that were correlated to all of the other characters were LA, MLW, LW1, LW2 and PL. However, the largest number of strong correlations was observed for LA (6) and MLW (5), while correlations were the weakest in traits related to leaf shape: FC, LA1 and LA2. A strong correlation among leaf shape traits was detected only between LA1 and LA2. No significant correlation was detected between LL and FC or LA1 and LA2 or between PMLW and the same three traits.

Observing the fruit morphological traits (Table S3), significant correlations were noted between all of the measured traits except WA. In total, seven strong correlations were detected, four of which refer to MA (ML, MMW, PMMW, MW90). In general, nut characteristics were less significantly correlated with other fruit traits.

Analysis between leaf and fruit traits showed 33 significant correlations (Table S4), all of which were classified as very weak or weak. No significant correlations were detected between MMW, NW and WA and any leaf traits. On the other hand, traits ML, PMMW and NL were significantly correlated to all of the leaf traits except LA1 and LA2, which showed no significant correlations with any of the fruit traits.

### 3.2. Leaf Phenotypic Traits

The results of the performed statistical analysis for leaf morphometric traits are shown in Table 1, by population and for all populations together. The average leaf length (LL) was 6.29 cm, whereas the average maximum blade width was 4.08 cm. Mean petiole length (PL) was 4.30 cm, and the overall mean leaf area, for all populations, was 18.38 cm^2^. The highest mean coefficient of variation was noted for the trait LW2 (37.40%), with the second highest noted for LA (36.45%). The lowest coefficients of variations were noted for both leaf angles measured, 4.44% and 6.50%, for LA1 and LA2, respectively.

The most variable population was P3, with the maximum values of coefficient of variation for seven out of ten measured traits (LA, LL, MLW, PMLW, LW1, PL, LA2). Furthermore, population P2 was characterised by the overall largest leaves, having the highest mean values of six leaf traits (LA, LL, MLW, LW1, LW2, PL). On the other hand, population P8 was characterised by the smallest leaves (LA, LL, MLW, PMLW, LW1, LW2, PL), as well as the narrowest leaf angles (LA1, LA2) and the most elongated leaf blade shape (FC). In addition, P8 was characterised by the most homogeneous values, i.e., the least variable leaf traits, with five traits having the minimum coefficient of variation values (LA, LL, MLW, LW1, LW2).

### 3.3. Fruit Phenotypic Traits

The results of the morphometric analysis of fruits have been presented on the overall sample, as well as on the individual population’s level (Table 2). The average mericarp area (MA), i.e., the area of the winged nutlet, on the overall level was 2.53 cm^2^, whereas the overall mean length (ML) was 3.18 cm. The average nut length (NL) came up to 1.23 cm, with a mean width (NW) of 0.63 cm. The angle enclosed by the two samaras, i.e., the two mericarps, had a mean value of 66.35°. The most variable trait was the single mericarp area (MA), with a coefficient of variation of 25.96%. The remaining fruit traits were characterised by prominently lower coefficients of variation, which ranged between 12.38% for mericarp length (ML) up to 19.88% for the angle enclosed by the two mericarps (WA).

Population P8 was characterised by having the lowest mean values for six of the measured traits: MA, ML, MMW, PMMW, MW90 and NL. In contrast, population P1 was defined by having the longest mericarp length (ML) and largest mericarp area (MA). In addition, maximum values were also found in population P6, for PMMW, NW and WA. The least variable population was P1, with lowest coefficient of variation values for four out of eight measured traits: MA, MMW, NW and WA. As the most variable population, P7 stands out, with four traits having the highest values of coefficient of variation (MA, ML, PMMW, NW).

### 3.4. Analysis of Variance—ANOVA

To determine the significance of differentiation by each individual trait, ANOVA analysis was performed. Both populations and individual maple shrubs/trees differed significantly when analysed leaf traits are considered (Table 3). For all analysed traits, between 50 and 75% of total variance could be ascribed to variance between leaves of the same individual, found as the error component. The smallest component of the variance could be ascribed to the interpopulation diversity, only 4.58–16.28% of total variance, with the overall lowest value of 4.58% found in LA1. The intrapopulation diversity had intermediate values, with a range of 16.49–36.64%.

Results of the performed ANOVA analysis for measured fruit traits can be found in detail in Table 4. The results of ANOVA analysis for fruit traits of Tatar maple showed significant differences between populations of Tatar maple, as well as between trees/shrubs of the same population, for all of the eight measured traits. The majority of measured traits had the largest proportion of variability assigned to the intrapopulation variability, around 50%. The proportions of variability assigned to the interpopulation component and the error component were similar, with only two traits, NW and PMNW, demonstrating notably larger proportions assigned to the error component.

### 3.5. Mantel Test

**Figure 3:**
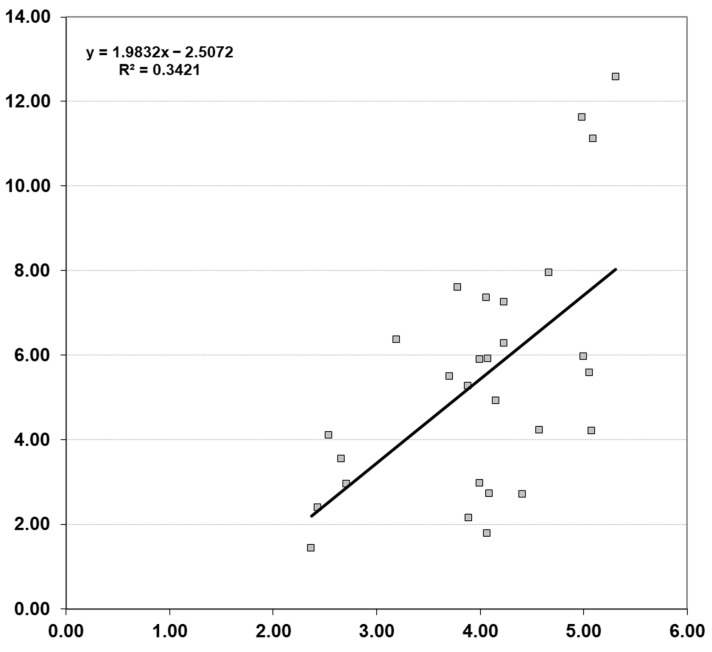
Isolation by distance (IBD) in Tatar maple populations. Scatter plot of simple Mantel test showing the relationship between geographic and leaf phenotypic distances (*r* = 0.585, *p* = 0.0092, R^2^ = 0.3421).

The results of the Mantel test are shown in Figure 3. Statistical significance was observed only between geographical and leaf morphological distances, which indicates that morphologically similar populations were also geographically closer. Linear regression of those distances was used to obtain a regression line, and the coefficient of determination accounted for R^2^ = 0.3421. Thus, 34.21% of leaf morphological variability among analysed populations could be explained by their geographic distance. On the other hand, a significant relationship between environmental distances between populations and their leaf (*r* = 0.407, *p* = 0.083) and fruit (*r* = 0.361, *p* = 0.140) morphological distances was not recorded. In addition, no statistically significant correlations between fruit morphological distances and geographical distances (*r* = 0.451, *p* = 0.098), nor between leaf and fruit morphological distances (*r* = 0.319, *p* = 0.149), were disclosed.

## 4. Discussion

Tatar maple is a poorly researched species, with no discernible morphological studies conducted so far. Even so, the data on leaf dimensions can be found in several written publications, with leaf size being reported to be between 6 and 10 cm long and 3 to 8 cm wide, with a petiole of about 2.5 cm [34,40,41]. These values are in accordance with those revealed in this study, albeit the petiole length of the populations presented here is much longer, on average 4.30 cm. This deviation in petiole length might be due to adjustments in phyllotaxy, directed towards better light capture efficiency [42], and due to the fact that plants tend to display longer petioles on more productive habitats [43]. Adjustments in the petiole length can also be stimulated for better nutrient transport efficiency and leaf support and protection [44]. Such adaptations in petiole length are enabled through differences in the total number of cells, individual cell size, or both [45]. In addition, the average mericarp length (ML) in this study was 3.18 cm, and it is slightly longer than the values reported by Bartha [34] and Idžojtić [46], with reported mericarp values of 2 to 3 cm.

Positive correlations between leaf/fruit size morphological traits, like leaf/fruit area, length and width, were noted in our research. Such a relation between named traits is expected and has been previously reported for woody plant species within *Populus* L. [47], *Theobroma* L. [13] and *Alnus* L. [48] species, as well as on a general level [49]. According to the latter, the length and width of leaves/fruits directly influence within-species variation of total leaf/fruit area with high accuracy. On the other hand, the relationship between leaf and fruit morphological traits is less studied. Fishler et al. [50] indicate that the leaf area is one of the main limiting factors in fruit growth, as greater photosynthetically active leaf area better supplies fruit growth, therefore determining its size. In our research, there was a very weak or weak correlation between leaf and fruit traits, few of which were statistically significant. Similar results were reported for *Eucommia ulmoides* Oliv. [51] and *Malania oleifera* Chun and S.K. Lee [52].

With regard to the coefficients of variability, they ranged from 4.44% to 36.45% for leaf traits. The high levels of variability found for leaf traits aren’t surprising, considering the importance of morphological adaptability of leaves for the survival of plants, since leaves are the key players in the interaction with the environment [53]. Leaves, as organs sensitive to changes in the environment, exhibit phenotypic variability as a response to stressors, which in turn can alter the primary morphogenesis [9]. Previous studies have even confirmed a global trend of leaf trait variation across individual species with broad and diverse biogeographical and environmental affinities [54,55]. This is true for numerous plant species, in accordance with various environmental factors, such as light availability [56,57,58], altitude [59,60,61] and relative humidity [62,63], and attests to the importance of leaf adaptations for survival and growth [53]. Unlike leaves, fruits had significantly lower coefficients of variation, within the range of 12.38% to 25.96%. The lower variability of fruit traits revealed in this research is not uncommon. Leaf traits have been continuously found to be more variable than those of fruits, for numerous species, such as *Pterocarpus erinaceus* Poir. [64], *Staphylea japonica* (Thunb.) Mabb. [65] and *Malus zumi* (Matsum.) Rehder [66]. This fact could be attributed to the pronounced influence of the environmental factors on the leaves as main assimilation organs [67], which favours greater variability.

The high levels of variability in leaf shape and size, as well as in the fruits of Tatar maple, were found on both levels, intra- and interpopulation. In both cases, intra-population variability was greater than inter-population variability. This result is generally consistent with that predicted by population genetics theory when the plant species are taken into consideration. In general, it is well known that woody species maintain more variation within populations than other types of species, such as annual and perennial herbaceous species, but have less variation among populations [17]. Woody species with large geographic ranges, outcrossing breeding systems and wind or animal-ingested seed dispersal have more genetic diversity within species and populations but less variation among populations than woody species with other combinations of traits. In addition, long-lived plant species with large ranges and high fecundities typically have large, stable populations. Such populations are resistant to chance fluctuations in gene or genotype frequencies and should maintain more variation than populations that experience large fluctuations in size.

When the results of the hierarchical variance analysis are considered, some differences in the distribution of the total variability can be noted when leaf and fruit variabilities are compared. For leaves, the greatest component of the total variability is assigned to the leaves’ variability within each tree, and, in the case of fruits, it is assigned to the variability of the individuals within populations. Furthermore, interpopulation variability was somewhat greater when measured fruit traits were compared to those measured on leaves. These different variance partitioning patterns suggest to us that different factors affect reproductive and vegetative traits. As already mentioned, reproductive traits can be considered more evolutionarily conserved than vegetative traits [68], and vegetative traits can be considered evolutionarily more “plastic” [69]. In general, leaf traits’ components of variability indicated greater influence of the environmental conditions and leaf placement on the shoots, as the error component was by far the most significant in the overall variability. In other words, leaves are the organs that respond to the heterogeneity of the environment stronger than fruits, possibly due to their significance in the overall adaptability and survival of the plants [70]. Fruit morphology, on the other hand, has been shown to be under stronger genetic influence, i.e., adaptive evolution, in numerous species, and as such is less likely to demonstrate high levels of variability [71,72,73]. Similar patterns of variability in leaf and fruit traits were found in other species as well: *Castanea sativa* Mill. [74,75,76,77], *Ulmus laevis* Pall [78] and *Sorbus domestica* L. [79].

In addition to lower levels of morphological variability, fruits analysed in this research showed no significant correlation to either isolation by distance (IBD) or isolation by environment (IBE). On the other hand, leaf morphology was found to have a significant correlation to IBD, with more distant populations demonstrating greater morphological differences. In marginal populations, both the specific environments and geographical distance can affect morphological variability, as those populations are often fragmented geographically and, due to varying environments, develop habitat-specific adaptations [80,81]. Such morphological differentiation of marginal populations has previously been noted in *Juniperus thurifera* L. [82] in northern Africa and *Pinus sylvestris* L. [83] in the western Mediterranean, with both species demonstrating significant morphological differences in their marginal populations, much like the populations analysed here. Being the marginal populations, they are younger [28] and were less likely to have had time for pronounced adaptation to local environments, which would have resulted in development of IBE. Additionally, though geographically isolated, they are found in relatively homogeneous pedological and climatological conditions, which could hinder ecological diversification. This lack of selection signature in leading-edge populations has been previously noted in younger, leading-edge populations of *Biscutella laevigata* L., which demonstrated a smaller degree of local adaptation than the trailing-edge populations of the same species, thus inhibiting the IBE [84].

## 5. Conclusions

Southeastern Europe represents the northwestern edge of Tatar maple natural distribution. In such marginal populations, both the specific environments and geographical distance can affect morphological variability, as those populations are often geographically and ecologically fragmented. Indeed, in this study, high levels of variability were found for leaf traits, attributed to their morphological adaptability and variability conditioned by environmental heterogeneity. Unlike leaves, fruits had significantly lower coefficients of variation. The high levels of morphological variability of Tatar maple were found at both intra- and interpopulation levels. In both cases, intra-population variability was greater than inter-population variability. Weak but significant correlations were found between some of the leaf and fruit characters. However, according to the Mantel test, the only significant correlation was found between geographical and leaf morphological distances , i.e., isolation-by-distance pattern. Overall, studied edge populations of Tatar maple are characterised by great morphological variability, which represents a coping mechanism that helps these populations in their response to the intensive selective pressures. In the future, such pressures will be even more frequent due to climate change, so northward expansion of the Tatar maple natural range could be expected.

## Figures and Tables

**Table 1:** Results of the descriptive statistical analysis for studied populations and leaf morphometric traits. Leaf morphometric traits analysed: LA—leaf area; LL—leaf length; MLW—maximum leaf width; PMLW—leaf length, measured from the leaf base to the point of maximum leaf width; LW1—leaf blade width at 50% of the leaf blade length; LW2—leaf blade width at 90% of the leaf blade length; PL—petiole length; FC—form coefficient; LA1—angle closed by the main leaf vein (the centre of the leaf blade) and the line connecting the leaf blade base to a set point on the leaf margin at 10% of the total leaf blade length; and LA2—angle closed by the main leaf vein (the centre of the leaf blade) and the line connecting the leaf blade base to a set point on the leaf margin at 25% of the total leaf blade length. Descriptive parameters: M—arithmetic mean and CV—coefficient of variation (%). Populations: P1—Odransko polje; P2—Lipovljani; P3—Veliki Grđevac; P4—Mali Grđevac; P5—Grubišno Polje; P6—Virovitica; P7—Požega; P8—Županja.

Trait	Descriptive Parameters	Population								Total
		P1	P2	P3	P4	P5	P6	P7	P8	
LA (cm^2^)	M	20.51	21.76	18.22	18.20	17.31	19.76	19.64	14.04	18.38
	CV	34.96	26.66	40.85	33.82	38.39	36.08	29.37	25.50	36.45
LL (cm)	M	6.70	6.75	6.30	6.26	6.08	6.36	6.49	5.76	6.29
	CV	18.59	14.00	20.27	16.06	18.28	17.85	14.20	13.76	17.64
MLW (cm)	M	4.28	4.55	3.96	4.07	3.95	4.28	4.29	3.56	4.08
	CV	20.72	15.73	23.18	19.71	21.36	20.10	17.41	14.68	20.70
PMLW (cm)	M	2.33	2.32	2.10	2.05	1.93	2.08	2.15	1.87	2.08
	CV	26.72	20.78	29.42	25.80	28.21	24.75	24.21	24.30	26.75
LW1 (cm)	M	3.73	4.00	3.50	3.58	3.40	3.75	3.69	2.98	3.54
	CV	19.84	17.17	22.98	20.51	22.60	20.75	17.65	15.18	21.56
LW2 (cm)	M	0.79	0.89	0.83	0.81	0.81	0.82	0.80	0.60	0.78
	CV	31.07	33.38	35.20	36.91	40.96	38.74	36.98	26.61	37.40
PL (cm)	M	4.63	5.30	4.05	3.99	4.28	4.56	4.69	3.63	4.30
	CV	28.75	22.31	33.60	30.87	28.44	29.91	32.85	25.94	31.47
FC	M	0.65	0.62	0.60	0.62	0.67	0.69	0.63	0.55	0.63
	CV	13.52	14.95	15.34	15.18	12.26	13.19	17.37	15.61	16.29
LA1 (°)	M	67.41	68.73	67.21	67.85	68.10	69.10	68.14	67.09	67.91
	CV	3.86	3.72	4.42	4.83	4.13	4.07	4.29	4.73	4.44
LA2 (°)	M	49.58	51.05	48.93	50.00	50.31	51.32	50.70	48.83	50.00
	CV	5.10	5.76	6.80	6.66	6.70	5.58	6.47	6.47	6.50

**Table 2:** Results of the descriptive statistical analysis for studied populations and fruit morphometric traits. Fruit morphometric traits analysed: MA—mericarp area; ML—mericarp length; MMW—maximum mericarp width; PMMW—mericarp length, measured from the mericarp base to the point of maximum mericarp width; NW90—mericarp width at 90% of fruit length; NL—nut length; NW—nut width; WA—wing angle. Descriptive parameters: M—arithmetic mean and CV—coefficient of variation (%). Populations: P1—Odransko polje; P2—Lipovljani; P3—Veliki Grđevac; P4—Mali Grđevac; P5—Grubišno Polje; P6—Virovitica; P7—Požega; P8—Županja.

Trait	Descriptive Parameters	Population								Total
		P1	P2	P3	P4	P5	P6	P7	P8	
MA (cm^2^)	M	2.96	2.30	2.59	2.45	2.58	2.80	2.93	1.87	2.53
	CV	16.47	21.14	22.38	16.96	22.67	19.96	27.24	26.43	25.96
ML (cm)	M	3.48	3.15	3.22	3.18	3.21	3.35	3.18	2.75	3.18
	CV	7.55	9.29	10.33	7.24	10.38	9.94	14.08	13.92	12.38
MMW (cm)	M	1.26	1.06	1.19	1.16	1.17	1.24	1.35	0.97	1.17
	CV	12.41	17.47	18.29	13.50	15.30	13.16	17.01	15.66	18.18
PMMW (cm)	M	2.43	2.26	2.30	2.30	2.29	2.44	2.26	1.98	2.27
	CV	9.85	11.95	13.79	7.83	11.13	9.61	14.91	14.03	13.33
NW90 (cm)	M	0.90	0.78	0.86	0.83	0.84	0.93	1.00	0.71	0.85
	CV	16.84	17.36	19.28	14.28	18.35	14.64	16.87	16.38	19.39
NL (cm)	M	1.30	1.25	1.31	1.24	1.19	1.28	1.26	1.03	1.23
	CV	9.89	8.80	10.43	8.54	8.33	8.15	12.87	13.59	12.67
NW (cm)	M	0.66	0.64	0.66	0.65	0.57	0.67	0.62	0.59	0.63
	CV	9.56	13.04	14.31	15.34	11.05	13.66	15.62	13.06	14.54
WA	M	59.67	71.56	70.75	66.99	55.02	73.35	65.97	64.13	66.35
	CV	15.13	15.94	20.90	19.89	20.31	16.23	17.76	16.75	19.88

**Table 3:** Hierarchical analysis of variance for leaf morphometric traits. Leaf morphometric traits analysed: LA—leaf area; LL—leaf length; MLW—maximum leaf width; PMLW—leaf length, measured from the leaf base to the point of maximum leaf width; LW1—leaf blade width at 50% of the leaf blade length; LW2—leaf blade width at 90% of the leaf blade length; PL—petiole length; FC—form coefficient; LA1—angle closed by the main leaf vein (the centre of the leaf blade) and the line connecting the leaf blade base to a set point on the leaf margin at 10% of the total leaf blade length; and LA2—angle closed by the main leaf vein (the centre of the leaf blade) and the line connecting the leaf blade base to a set point on the leaf margin at 25% of the total leaf blade length. n.s. not significant at *p* > 0.05; * significant at 0.01 < *p* < 0.05; ** significant at 0.001 < *p* < 0.01; *** significant at *p* < 0.001.

Trait	Variance Component	df	% Variation	F	*p*
LA	Among populations	7	11.02	7.68	***
	Within populations	89	17.40	5.87	***
	Error		71.58		
LL	Among populations	7	6.69	5.03	***
	Within populations	89	16.49	5.29	***
	Error		76.82		
MLW	Among populations	7	11.34	8.12	***
	Within populations	89	16.51	5.58	***
	Error		72.15		
PMLW	Among populations	7	6.10	4.40	***
	Within populations	89	17.22	5.49	***
	Error		76.68		
LW1	Among populations	7	13.39	7.97	***
	Within populations	89	21.69	7.69	***
	Error		64.92		
LW2	Among populations	7	7.15	4.11	***
	Within populations	89	26.81	9.15	***
	Error		66.03		
PL	Among populations	7	13.37	7.57	***
	Within populations	89	20.75	7.30	***
	Error		65.88		
FC	Among populations	7	16.28	6.75	***
	Within populations	89	36.64	16.60	***
	Error		47.08		
LA1	Among populations	7	4.58	2.60	**
	Within populations	89	32.61	11.40	***
	Error		62.81		
LA2	Among populations	7	6.52	3.43	**
	Within populations	89	31.19	11.04	***
	Error		62.29		

**Table 4:** Hierarchical analysis of variance for fruit morphometric traits. Fruit morphometric traits analysed: MA—mericarp area; ML—mericarp length; MMW—maximum mericarp width; PMMW—mericarp length, measured from the mericarp base to the point of maximum mericarp width; NW90—mericarp width at 90% of fruit length; NL—nut length; NW—nut width; WA—wing angle. n.s. not significant at *p* > 0.05; * significant at 0.01 < *p* < 0.05; ** significant at 0.001 < *p* < 0.01; *** significant at *p* < 0.001.

Trait	Variance Component	df	% Variation	F	*p*
MA	Among populations	7	25.87	7.25	***
	Within populations	89	51.59	46.83	***
	Error		22.54		
ML	Among populations	7	24.42	7.32	***
	Within populations	89	49.72	39.52	***
	Error		25.86		
MMW	Among populations	7	25.87	6.90	***
	Within populations	89	52.64	50.00	***
	Error		21.49		
PMMW	Among populations	7	19.68	7.23	***
	Within populations	89	41.16	22.07	***
	Error		39.16		
NW90	Among populations	7	23.65	7.73	***
	Within populations	89	41.83	25.24	***
	Error		34.52		
NL	Among populations	7	31.50	9.86	***
	Within populations	89	47.47	46.21	***
	Error		21.03		
NW	Among populations	7	11.34	3.40	**
	Within populations	89	54.71	33.25	***
	Error		33.95		
WA	Among populations	7	15.74	3.84	**
	Within populations	89	62.17	57.24	***
	Error		22.09		

## Data Availability

Data are contained within the article or Supplementary Material.

## Supplementary Materials

The following supporting information can be downloaded at https://www.mdpi.com/article/10.3390/plants13020320/s1: Table S1: Geographic coordinates, altitudes, bioclimatic variables and number of samples for eight studied *Acer tataricum* L. subsp. *tataricum* populations. Bioclimatic variables: BIO1 (Annual Mean Temperature); BIO2 (Mean Diurnal Range (Mean of monthly (max temp—min temp)); BIO3 (Isothermality (BIO2/BIO7) (×100)); BIO4 (Temperature Seasonality (standard deviation ×100)); BIO5 (Max Temperature of Warmest Month); BIO6 (Min Temperature of Coldest Month); BIO7 (Temperature Annual Range (BIO5-BIO6)); BIO8 (Mean Temperature of Wettest Quarter); BIO9 (Mean Temperature of Driest Quarter); BIO10 (Mean Temperature of Warmest Quarter); BIO11 (Mean Temperature of Coldest Quarter); BIO12 (Annual Precipitation); BIO13 (Precipitation of Wettest Month); BIO14 (Precipitation of Driest Month); BIO15 (Precipitation Seasonality (Coefficient of Variation)); BIO16 (Precipitation of Wettest Quarter); BIO17 (Precipitation of Driest Quarter); BIO18 (Precipitation of Warmest Quarter); BIO19 (Precipitation of Coldest Quarter). Populations: P1–Odransko polje; P2–Lipovljani; P3–Veliki Grđevac; P4–Mali Grđevac; P5–Grubišno Polje; P6–Virovitica; P7–Požega; P8–Županja; Table S2: Pearson’s correlation coefficients between analysed leaves’ traits. Leaves’ morphometric traits acronyms: leaf area (LA); leaf length (LL); maximum leaf width (MLW); leaf length, measured from the leaf base to the point of maximum leaf width (PMLW); leaf blade width at 50% (LW1) and 90% of leaf blade length (LW2); petiole length (PL); form coefficient (FC); angles enclosed by the main leaf vein (the centre of the leaf blade) and the line connecting the leaf blade base to a set point on the leaf margin, at 10% (LA10) and 25% (LA25). n.s. not significant at *p* > 0.05; * significant at 0.01 < *p* < 0.05; ** significant at 0.001 < *p* < 0.01; *** significant at *p* < 0.001; Table S3: Pearson’s correlation coefficients between analysed fruit traits. Fruit morphometric traits acronyms: mericarp area (MA); mericarp length (ML); maximum mericarp width (MMW); length of the mericarp, measured from the basis to the point of maximum width (PMMW); width of mericarp at 90% of mericarp’s length (MW90); nut length (NL); nut width (NW); angle enclosed by the wings (WA). n.s. not significant at *p* > 0.05; * significant at 0.01 < *p* < 0.05; ** significant at 0.001 < *p* < 0.01; *** significant at *p* < 0.001; Table S4: Pearson’s correlation coefficients between analysed leaves and fruit traits. Leaves’ morphometric traits acronyms: leaf area (LA); leaf length (LL); maximum leaf width (MLW); leaf length, measured from the leaf base to the point of maximum leaf width (PMLW); leaf blade width at 50% (LW1) and 90% of leaf blade length (LW2); petiole length (PL); form coefficient (FC); angles enclosed by the main leaf vein (the centre of the leaf blade) and the line connecting the leaf blade base to a set point on the leaf margin, at 10% (LA10) and 25% (LA25). Fruit morphometric traits acronyms: mericarp area (MA); mericarp length (ML); maximum mericarp width (MMW); length of the mericarp, measured from the basis to the point of maximum width (PMMW); width of the mericarp at 90% of the mericarp’s length (MW90); nut length (NL); nut width (NW); angle enclosed by the wings (WA). n.s. not significant at *p* > 0.05; * significant at 0.01 < *p* < 0.05; ** significant at 0.001 < *p* < 0.01; *** significant at *p* < 0.001.
