# Supplementary material for: Patterns of Leaf and Fruit Morphological Variation in Marginal Populations of Acer tataricum L. subsp. tataricum
Source: Plants (Basel). 2024 Jan 21;13(2):320. doi: 10.3390/plants13020320 (PMC10818317; doi:10.3390/plants13020320)
Supplement: Supplementary file 1 [file plants-13-00320-s001.zip › plants-2781729-supplementary.pdf]

Table S1: Geographic coordinates, altitudes, bioclimatic variables and number of samples for eight studied *Acer tataricum* L. subsp. *tataricum* populations. Bioclimatic variables: BIO1 (Annual Mean Temperature); BIO2 (Mean Diurnal Range (Mean of monthly (max temp—min temp)); BIO3 (Iso-thermality (BIO2/BIO7) (×100)); BIO4 (Temperature Seasonality (standard deviation ×100)); BIO5 (Max Temperature of Warmest Month); BIO6 (Min Temperature of Coldest Month); BIO7 (Temperature Annual Range (BIO5-BIO6)); BIO8 (Mean Temperature of Wettest Quarter); BIO9 (Mean Temperature of Driest Quarter); BIO10 (Mean Temperature of Warmest Quarter); BIO11 (Mean Temperature of Coldest Quarter); BIO12 (Annual Precipitation); BIO13 (Precipitation of Wettest Month); BIO14 (Precipitation of Driest Month); BIO15 (Precipitation Seasonality (Coefficient of Variation)); BIO16 (Precipitation of Wettest Quarter); BIO17 (Precipitation of Driest Quarter); BIO18 (Precipitation of Warmest Quarter); BIO19 (Precipitation of Coldest Quarter). Populations: P1–Odransko polje; P2–Lipovljani; P3–Veliki Grđevac; P4–Mali Grđevac; P5–Grubišno Polje; P6–Virovitica; P7–Požega; P8–Županja.

Pop.	N	Longitude	Latitude	Altitude	Bioclimatic variables																		
					1	2	3	4	5	6	7	8	9	10	11	12	13	14	15	16	17	18	19
P1	10	45.550000	16.375278	99	11.2	9.3	29.9	755.4	25.5	-5.7	31.2	20.2	3.3	20.2	1.5	893	90	52	17.8	255	165	255	174
P2	10	45.374722	16.829444	105	11.2	10.0	32.3	731.0	25.5	-5.4	30.9	20.0	3.6	20.0	1.9	918	93	54	16.1	263	175	263	183
P3	15	45.730556	17.071389	119	11.1	9.8	31.5	762.6	25.4	-5.9	31.3	20.3	3.2	20.3	1.2	842	92	46	19.7	255	153	255	159
P4	12	45.798056	17.182222	144	11.0	9.5	31.3	734.7	24.9	-5.6	30.5	19.8	3.3	19.8	1.5	818	91	44	20.8	252	146	252	154
P5	10	45.707500	17.230556	136	11.1	9.6	31.2	756.0	25.2	-5.6	30.8	20.3	3.3	20.3	1.4	848	95	46	20.4	260	154	260	159
P6	15	45.803611	17.366389	138	11.1	8.9	30.3	744.8	24.6	-4.8	29.4	20.1	3.4	20.1	1.6	797	92	42	22.1	249	140	249	148
P7	10	45.337778	17.571667	222	10.9	10.3	32.6	757.3	25.3	-6.3	31.6	20.0	3.0	20.0	1.3	860	100	50	19.4	259	160	259	171
P8	15	44.973611	18.817222	85	11.2	10.3	32.9	775.3	25.7	-5.6	31.3	19.2	3.2	20.6	1.5	733	94	43	22.7	234	135	234	147

Table S2: Pearson's correlation coefficients between analysed leaves' traits. Leaves' morphometric traits acronyms: leaf area (LA); leaf length (LL); maximum leaf width (MLW); leaf length, measured from the leaf base to the point of maximum leaf width (PMLW); leaf blade width at 50% (LW1) and 90% of leaf blade length (LW2); petiole length (PL); form coefficient (FC); angles enclosed by the main leaf vein (the centre of the leaf blade) and the line connecting the leaf blade base to a set point on the leaf margin, at 10% (LA10) and 25% (LA25). n.s. not significant at $p > 0.05$; * significant at $0.01 < p < 0.05$; ** significant at $0.001 < p < 0.01$; *** significant at $p < 0.001$

	LA	LL	MLW	PMLW	LW1	LW2	PL	FC	LA1	LA2
LA		***	***	***	***	***	***	**	**	**
LL	0.9010		***	***	***	***	***	n.s.	n.s.	n.s.
MLW	0.9543	0.7769		***	***	***	***	***	***	***
PMLW	0.7224	0.7436	0.6903		***	***	***	n.s.	n.s.	n.s.
LW1	0.9192	0.7193	0.9541	0.7535		***	***	***	***	***
LW2	0.7560	0.5276	0.7266	0.4884	0.7782		***	***	***	***
PL	0.7914	0.6631	0.7883	0.4503	0.6915	0.6290		***	***	***
FC	0.3375	0.0785	0.3558	-0.0397	0.3610	0.5438	0.4260		***	***
LA1	0.3021	-0.0774	0.4835	-0.1018	0.4456	0.3871	0.4340	0.5504		***
LA2	0.3253	-0.0748	0.5428	-0.0223	0.5037	0.4276	0.4437	0.5655	0.9402	

Table S3: Pearson's correlation coefficients between analysed fruit traits. Fruit morphometric traits acronyms: mericarp area (MA); mericarp length (ML); maximum mericarp width (MMW); length of the mericarp, measured from the basis to the point of maximum width (PMMW); width of mericarp at 90% of mericarp's length (MW90); nut length (NL); nut width (NW); angle enclosed by the wings (WA). n.s. not significant at $p > 0.05$; * significant at $0.01 < p < 0.05$; ** significant at $0.001 < p < 0.01$; *** significant at $p < 0.001$.

	MA	ML	MMW	PMMW	MW90	NL	NW	WA
MA		***	***	***	***	***	**	n.s
ML	0.882		***	***	***	***	***	n.s
MMW	0.935	0.702		***	***	***	*	n.s
PMMW	0.754	0.908	0.585		***	***	***	n.s
MW90	0.831	0.596	0.893	0.633		***	**	n.s
NL	0.618	0.698	0.505	0.686	0.491		***	n.s
NW	0.323	0.420	0.249	0.503	0.321	0.637		n.s
WA	-0.065	-0.073	-0.053	-0.021	-0.018	-0.081	-0.058	

Table S4: Pearson's correlation coefficients between analysed leaves and fruit traits. Leaves' morphometric traits acronyms: leaf area (LA); leaf length (LL); maximum leaf width (MLW); leaf length, measured from the leaf base to the point of maximum leaf width (PMLW); leaf blade width at 50% (LW1) and 90% of leaf blade length (LW2); petiole length (PL); form coefficient (FC); angles enclosed by the main leaf vein (the centre of the leaf blade) and the line connecting the leaf blade base to a set point on the leaf margin, at 10% (LA10) and 25% (LA25). Fruit morphometric traits acronyms: mericarp area (MA); mericarp length (ML); maximum mericarp width (MMW); length of the mericarp, measured from the basis to the point of maximum width (PMMW); width of the mericarp at 90% of the mericarp's length (MW90); nut length (NL); nut width (NW); angle enclosed by the wings (WA). n.s. not significant at $p > 0.05$; * significant at $0.01 < p < 0.05$; ** significant at $0.001 < p < 0.01$; *** significant at $p < 0.001$.

	LA	LL	MLW	PMLW	LW1	LW2	PL	FC	LA1	LA2
MA	0.274**	0.249*	0.229*	0.139 n.s.	0.252*	0.237*	0.218*	0.249*	0.027 n.s.	0.063 n.s.
ML	0.337**	0.300**	0.289**	0.258*	0.342**	0.306**	0.268**	0.292**	0.038 n.s.	0.082 n.s.
MMW	0.190 n.s.	0.169 n.s.	0.166 n.s.	0.062 n.s.	0.179 n.s.	0.150 n.s.	0.128 n.s.	0.160 n.s.	0.014 n.s.	0.055 n.s.
PMMW	0.338**	0.325**	0.301**	0.302**	0.340**	0.250*	0.252*	0.288**	-0.005 n.s.	0.063 n.s.
MW90	0.206*	0.205*	0.189 n.s.	0.097 n.s.	0.174 n.s.	0.097 n.s.	0.136 n.s.	0.172 n.s.	-0.004 n.s.	0.049 n.s.
NL	0.384***	0.366***	0.303**	0.226*	0.349**	0.374***	0.263*	0.340**	0.023 n.s.	0.047 n.s.
NW	0.190 n.s.	0.172 n.s.	0.164 n.s.	0.070 n.s.	0.168 n.s.	0.130 n.s.	0.036 n.s.	0.188 n.s.	0.060 n.s.	0.079 n.s.
WA	0.117 n.s.	0.087 n.s.	0.134 n.s.	0.112 n.s.	0.159 n.s.	0.093 n.s.	0.127 n.s.	0.021 n.s.	0.096 n.s.	0.067 n.s.
